# Hepatoprotective Activity of the Ethanolic Extract of* Polygonum multiflorum* Thunb. against Oxidative Stress-Induced Liver Injury

**DOI:** 10.1155/2018/4130307

**Published:** 2018-10-17

**Authors:** En-Yuan Lin, Amarzaya Chagnaadorj, Shyh-Jer Huang, Ching-Chiung Wang, Yung-Hsiao Chiang, Chao-Wen Cheng

**Affiliations:** ^1^Graduate Institute of Clinical Medicine, College of Medicine, Taipei Medical University, Taipei, Taiwan; ^2^Division of Neurosurgery, Department of Surgery, Taiwan Adventist Hospital, Taipei, Taiwan; ^3^Department of Internal Medicine, Mongolian National University of Medical Sciences, Ulaanbaatar, Mongolia; ^4^Skin Institute, Hualien Tzu Chi Hospital, Hualien, Taiwan; ^5^Graduate Institute of Pharmacognosy, College of Pharmacy, Taipei Medical University, Taipei, Taiwan; ^6^School of Pharmacy, College of Pharmacy, Taipei Medical University, Taipei, Taiwan; ^7^Traditional Herbal Medicine Research Center, Taipei Medical University Hospital, Taipei Medical University, Taipei, Taiwan; ^8^Department of Neurosurgery, Taipei Medical University Hospital, Taipei Medical University, Taipei, Taiwan

## Abstract

Oxidative stress is an important pathological mechanism in various liver diseases.* Polygonum multiflorum* Thunb. (PM) can be used for the treatment of diseases associated with aging, hyperlipidemia, and oxidative stress in traditional Chinese medicine. In this study, we examined the hepatoprotective effects of the ethanolic extract of PM (PME) in in vitro and in vivo models. The PME induced expression of antioxidant-response-element- (ARE-) related genes in HepG2 cells showed a dose-dependent manner. Pretreatment of HepG2 cell with PME suppressed H_2_O_2_- and acetaminophen- (APAP-) induced cellular reactive oxygen species (ROS) generation and cytotoxicity. In APAP-induced mouse liver injury, pretreatment with PME also showed ability to increase the survival rate and reduce the severity of liver injury. Treatment with PME attenuated bile duct ligation-induced extrahepatic cholestatic liver injury and further increased multidrug resistance protein 4 (MRP4) and reduced organic anion-transporting polypeptide (OATP) expression. Furthermore, increased nuclear translocation of the nuclear factor erythroid 2-related factor 2 (Nrf2) was observed after treatment with PME in both in vivo models. In conclusion, the current study showed the hepatoprotective activity of PME by regulating the redox state in liver injury through Nrf2 activation and controlling hepatic bile acid homeostasis in obstructive cholestasis, through bile acid transporter expression modulation.

## 1. Introduction

Oxidative stress is widely recognized as a detrimental pathological mechanism for the initiation and progression of various liver diseases. Reactive oxygen species (ROS) are mainly generated by cytochrome P450 enzymes in the mitochondria of hepatocytes. When excess ROS are generated, they interact with proteins, DNA, and lipids, resulting in cell injury. In the course of liver diseases, either from inflammatory or metabolic insults, disruption of the balance between oxidant and antioxidant mechanisms is commonly observed [1, 2]. The abilities of the transcription factor, nuclear factor erythroid 2-related factor 2 (Nrf2), to increase the expressions of antioxidant proteins and suppress oxidative stress-related injury have been extensively studied [3, 4]. Therefore, Nrf2-activating regimens may be used for liver diseases.

*Polygonum multiflorum* Thunb. (PM) has long been used in traditional Chinese medicine to treat diseases associated with aging, hyperlipidemia, and oxidative stress. It can be used in two forms for different purposes. Raw PM is mainly used for detoxification. Processed PM is used as a tonic to reduce blood lipids and against arteriosclerosis- and aging-related symptoms [5]. The crude water extract of PM exhibited protective abilities against carbon tetrachloride-induced rat hepatotoxicity [6]. In addition, the active ingredients of PM, including polysaccharides [7], 2,3,5,4′-tetrahydroxystilbene-2-O-*β*-d-glucoside (THSG) [8], and emodin [9], also exhibited antioxidant activities. This evidence supports the potential ability of PM to nourish liver functions. However, adverse hepatic effects, including jaundice, fatigue, anorexia, and yellow or tawny urine, were consistently reported with PM use [5].

Actually, hundreds of compounds have been isolated from PM with different processing procedures that alter the properties and functions of remedies; so, a standardized processing procedure may be favorable for managing PM use. In acute toxicity screening using zebrafish embryos, PM extracted with different concentrations of an ethanolic solvent showed decreasing toxicity for 70% *≅* 95% > 50% > 30% > water [10]. When considering the solubility of active constituents, PM extracted with 50% ethanol (PME) retained its therapeutic activities while reducing potential toxicities. The current study examined the activities of PME against redox state-related liver injury using both in vitro and in vivo models. Cellular oxidative stress was created by exogenous treatment with H_2_O_2_ and acetaminophen (APAP) in HepG2 cells, while animal models of APAP-induced (xenobiotic-induced) liver injury and common bile-duct-ligation- (BDL-) induced extrahepatic cholestatic liver injury were used. In addition, potential modulating activities of PME on Nrf2 activation and expressions of bile acid channels were also addressed.

## 2. Materials and Methods

### 2.1. Preparation of PME

After identification, PM root was dissected into small pieces, immersed in 50% ethanol (1:10), and refluxed twice for 2 h at 65°C. Thereafter, ethanol was removed and freeze dried to yield PME powder containing approximately 34.09% of the original sample (w/w) [8].

### 2.2. Cell Culture

HepG2 cells were maintained in Dulbecco's modified Eagle's medium and F12 medium with 5% fetal bovine serum. Before every in vitro experiment, cells were seeded for 24 h and thereafter placed in serum-free medium for 12 h. All treatments proceeded in serum-free medium.

### 2.3. Cell Viability

HepG2 cells were seeded in a 96-well plate (1 × 10^4^ cells/well), the indicated concentrations of PME were added for 48 h, and the cell viability was determined using a One Solution Cell Proliferation Assay (MTS) (Promega, Madison, WI).

### 2.4. Cytotoxicity Assays

HepG2 cells were seeded in 24-well plates (1 × 10^5^ cells/well), incubated with or without 100 *μ*g/mL PME for 6 h, and further incubated with or without 10 mM APAP for 48 h. The supernatant was harvested, and CytoTox 96® Non-Radioactive Cytotoxicity Assay (LDH) (Promega) was used to determine the cell cytotoxicity.

### 2.5. Measurement of Intracellular ROS Production

Intracellular ROS production was monitored using two permeable fluorescence dyes: a chloromethyl derivative of 2′,7′-dichlorodihydrofluorescein diacetate (CM-H_2_DCFDA) and dihydroethidium (DHE). CM-H_2_DCFDA detected oxidation through hydroxides, hydrogen peroxides, and hydroxyl radicals, whereas DHE selectively detected superoxide anions [11].

For CM-H_2_DCFDA staining, HepG2 cells were cultured in a 96-well black-plate and incubated overnight. Cells were pretreated with or without 100 *μ*g/mL PME for 6 h, rinsed with Hank's balanced salt solution (HBSS) twice, and incubated with 10 *μ*M CM-H_2_DCFDA for 30 min in the dark. Subsequently, cells were washed with HBSS twice and incubated with 50 *μ*M H_2_O_2_, and the kinetic intensity was measured every hour.

For DHE staining, HepG2 cells were seeded in 24-well plates (1 × 10^5^ cells/well) and incubated for 3 h with or without 10 mM APAP after pretreatment with or without PME for 6 h. Cells were then trypsinized and resuspended in phosphate buffered saline with DHE working solution (100:1) at room temperature for 20 min in the dark and analyzed using a Muse® Cell Analyzer (Merck-Millipore, Billerica, MA). The numbers of ROS(+) and ROS(−) cells were presented as percentages of the total number of stained cells.

### 2.6. Messenger mRNA Analysis

An mRNA analysis was performed as previously described [8]. Primers applied in this study are listed in Table 1.

### 2.7. Western Blotting

Nuclear and cytoplasmic protein extracts were harvested from HepG2 cells treated with or without PME (100 *μ*g/mL) for 6 h and were prepared as described [12]. Protein samples (20 *μ*g) were separated using electrophoresis on a 4%–20% gradient gel prepared by a TOOLS HR Gradient gel solution (TOOLS Biotech, New Taipei City, Taiwan) and transferred to a polyvinylidene difluoride transfer membrane (Immunobilon-P, Millipore, Bedford, MA). Subsequently, the membrane was blocked using 2% bovine serum albumin and then incubated with an Nrf2 primary antibody (1:1000, ProteinTech, Chicago, IL) or lamin *β*1 (1:1000, Abcam, Cambridge, MA) and *α*-tubulin (1:1000 GeneTex, Irvine, CA) at 4°C overnight. After incubating with a secondary antibody, the Immobilon™ Western Chemiluminescent HRP Substrate (Merck-Millipore) was used to detect reactive protein signals.

### 2.8. Animal Models

Male ICR mice aged 8–10 weeks were purchased from LASCO (BioLASCO Taiwan, Taipei, Taiwan). All the mice were housed at the animal center of Taipei Medical University in a temperature-, light-, and humidity-controlled environment that is accredited by the animal center. The animal study was approved by the Animal Experimentation Ethics Committee of Taipei Medical University. Animals were sacrificed at indicated time points. Upon sacrifice, the mice were deeply anesthetized by intraperitoneal injection of Zoletil (50 mg/kg; Virbac Laboratory, France), and blood samples were obtained from retro-orbital venous plexus. Plasma was analyzed for clinical biochemical parameters using a VetTest Chemistry Analyzer. Livers were perfused with HBSS in situ through the portal vein, and left lateral lobes were sliced into approximately 3-mm-thick pieces and collected for mRNA expression and immunohistochemical (IHC) staining.

To assess APAP-induced mouse hepatotoxicity, the mice were separated into control (*n *= 3), APAP (*n *= 10), and PME+APAP (*n *= 10) groups. The mice were fasted for 16 h with free access to water before an intraperitoneal injection of saline or APAP (400 mg/kg). The PME+APAP group of mice were pretreated with 120 mg/kg PME for three consecutive days by oral gavage (o.g.) before administrating a single injection of APAP. The conditions of the mice were constantly monitored, and they were sacrificed at 6 h after APAP injection or when a moribund state was noted.

For surgical BDL-induced mouse extrahepatic cholestatic liver injury, the mice were separated into sham-operated (*n*=3), BDL (*n*=5), and BDL+PME (*n *= 5) groups. The BDL and sham-operated mice were performed as previously described [13]. The BDL+PME group mice were treated with 120 mg/kg PME by o.g. 6 h after BDL was performed and were then treated every day for 14 days.

### 2.9. IHC Staining

Paraffin-embedded liver tissues were sliced into 3-*μ*m sections for hematoxylin and eosin staining. IHC staining with an anti-Nrf2 antibody (1:100, ProteinTech) was performed using the avidin–biotin immunoperoxidase method as previously described [8].

### 2.10. Statistical Analysis

All results are presented as the mean ± standard error. Unpaired Student *t* testing was used to compare the two groups. One-way analysis of variance with Tukey post hoc analysis was used for determining differences between multiple groups, and* p *< 0.05 was considered statistically different.

## 3. Results

### 3.1. PME Induced Activation of the Nrf2 Pathway and Suppressed ROS Production in HepG2 Cells

**Figure 1:**
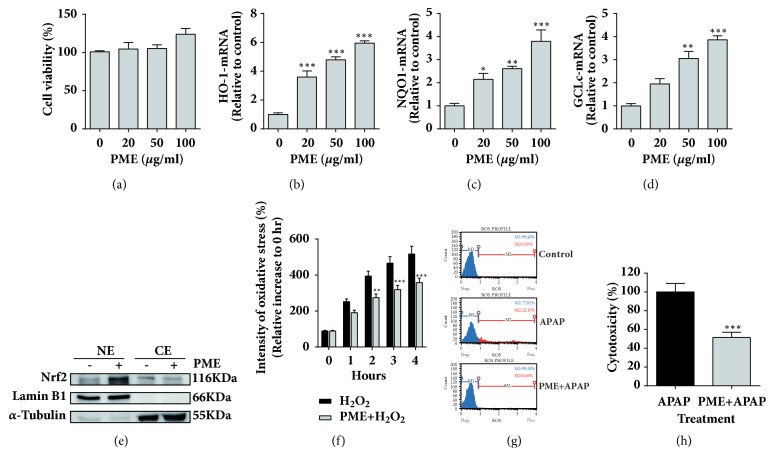
**PME-induced activation of the Nrf2 pathway and suppression of ROS production in HepG2 cells**. (a) Cell viability after treatment with various concentrations of PME for 48 h was detected by an MTS assay. (b–d) The mRNA expression levels of HO-1, NQO1, and GCLc at 6 h after treatment with various concentrations of PME. (e) Treatment with 100 *μ*g/mL PME for 6 h increased the Nrf2 levels in the nuclear fraction (NE), whereas the treatment decreased the levels in the cellular fraction (CE). Lamin B1 and *α*-tubulin were used as loading controls for NE and CE, respectively. (f) Pretreatment with 100 *μ*g/mL PME reduced 50 mM H_2_O_2_-induced cellular ROS generation. (g) Pretreatment with 100 *μ*g/mL PME reduced 10 mM APAP-induced increase in superoxide anion production (M2 population). (h) Pretreatment with 100 *μ*g/mL PME reduced 10 mM APAP-induced cytotoxicity. Data are expressed as [(OD_Detected_ − OD_Control_)/(OD_APAP_ − OD_Control_)] × 100%. *∗ p < 0.05, ∗∗ p <0.01; ∗∗∗ p < 0.001* compared with the control group.

Up to 100 *μ*g/mL PME, no significant negative effect on HepG2 cell viability was observed (Figure 1(a)). PME treatment for 6 h upregulated the mRNA expression of genes downstream of Nrf2, including heme oxygenase-1 (HO-1), NAD(P)H:quinone oxidoreductase (NQO1), and the glutamate–cysteine ligase catalytic subunit (GCLc), in a dose-dependent manner (Figures 1(b)–1(d)). Moreover, PME induced Nrf2 nuclear translocation (Figure 1(e)). We further examined the protective effects of PME against ROS production in HepG2 cells. PME pretreatment not only suppressed H_2_O_2_-induced intracellular ROS generation but also reduced APAP-mediated superoxide anion production and cytotoxicity (Figures 1(f)–1(h)). This evidence suggested that PME could induce Nrf2 pathway activation, suppress ROS production, and relieve APAP-mediated cytotoxicity in vitro.

### 3.2. PME Protected against APAP-Induced Mouse Hepatotoxicity

**Figure 2:**
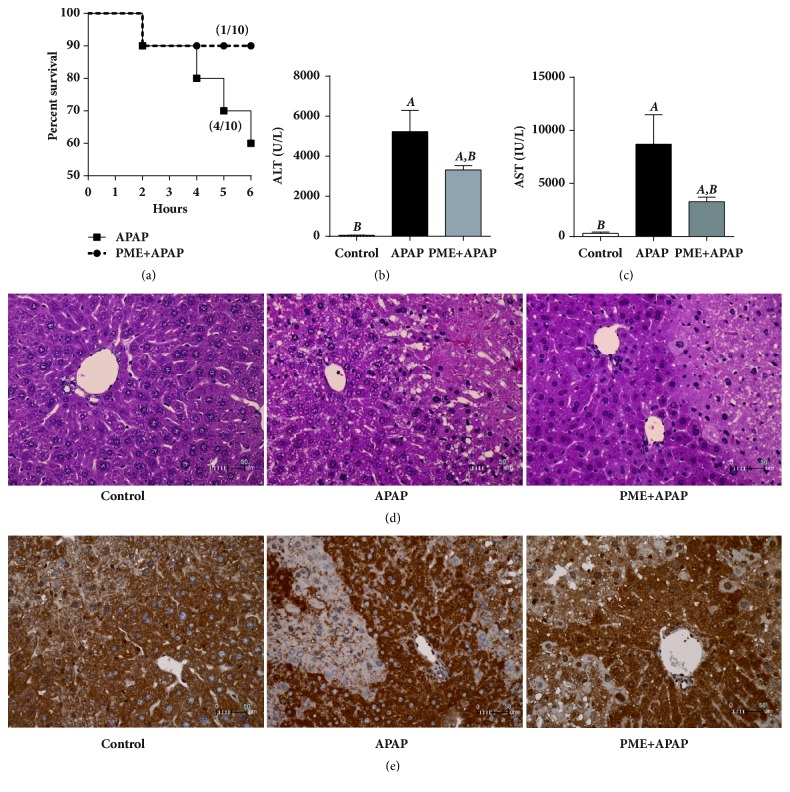
**Effect of PME against APAP-induced mouse hepatotoxicity**. Mortality (a), plasma ALT (b), and AST (c) levels were measured 6 h after the administration of water (control) or APAP. ^A^* p* < 0.05 compared with the control group; ^B^* p* < 0.05 compared with the APAP group;* n *= 3 in the control group;* n *= 10 each in the APAP and PME+APAP groups. Representative liver sections stained with hematoxylin and eosin (d) and anti-Nrf2 antibody (e) from untreated mice (left) and APAP-treated mice pretreated with either water (middle) or PME (right).

We further elucidated the preventive activities of PME in an animal model of APAP-induced liver injury. Four of the 10 mice treated with a single dose of 400 mg/kg APAP for 6 h died. By contrast only one of the 10 mice in the PME+APAP group died (Figure 2). The plasma alanine aminotransferase (ALT) and aspartate aminotransferase (AST) levels significantly increased in the APAP-treated mice but significantly decreased in the PME-pretreated mice (Figures 2(b) and 2(c)). The APAP-treated mice exhibited hepatic hemorrhage and diffuse massive hepatic cell necrosis, but PME-pretreated mice exhibited protective effects against hepatic hemorrhage and the size of the cell death region (Figure 2(d)). In addition, Nrf2 nuclear translocation increased in the PME+APAP group, whereas it decreased in the APAP and control groups (Figure 2(e)). These data indicated that PME can induce Nrf2 activation and prevent APAP-induced mouse hepatotoxicity.

### 3.3. PME Relieved Surgical BDL-Induced Mouse Extrahepatic Cholestatic Liver Injury

**Figure 3:**
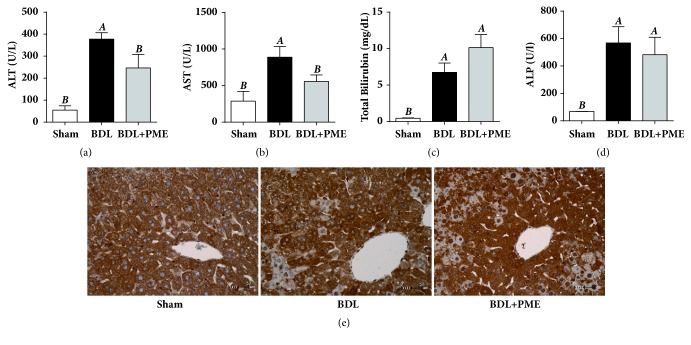
**PME relieved surgical BDL-induced mouse extrahepatic cholestatic liver injury**. Plasma ALT (a), AST (b), total bilirubin (c), and ALP (d) levels were measured 10 days after sham or BDL. ^A^* p* < 0.05 compared with the sham group; ^B^* p* < 0.05 compared with the BDL group;* n *= 3 in the sham group;* n *= 5 each in the BDL and BDL+PME groups. Representative liver sections stained with anti-Nrf2 antibody (e) from sham-operated mice (left) and BDL-treated mice treated with either water (middle) or PME (right).

Subsequently, the therapeutic activities of PME were assessed by surgical BDL-induced extrahepatic cholestatic liver injury in mice. On day 14 after surgery, the ALT, AST, total bilirubin, and alkaline phosphatase (ALP) levels were elevated in the plasma of BDL mice compared with sham-operated mice. Daily PME treatment significantly attenuated the increased ALT and AST levels in BDL mice but had no effect on the total bilirubin or ALP levels (Figures 3(a)–3(d)). Compared with the BDL and sham-operated groups, the PME+BDL group revealed distinctive Nrf2 nuclear translocation, which was indicated by an increased number of nuclear Nrf2 positive hepatocytes around hepatic lobule (Figure 3(e)). In addition to the preventive activities against APAP-induced liver injury, PME also ameliorated BDL-induced extrahepatic cholestatic liver injury.

**Figure 4:**
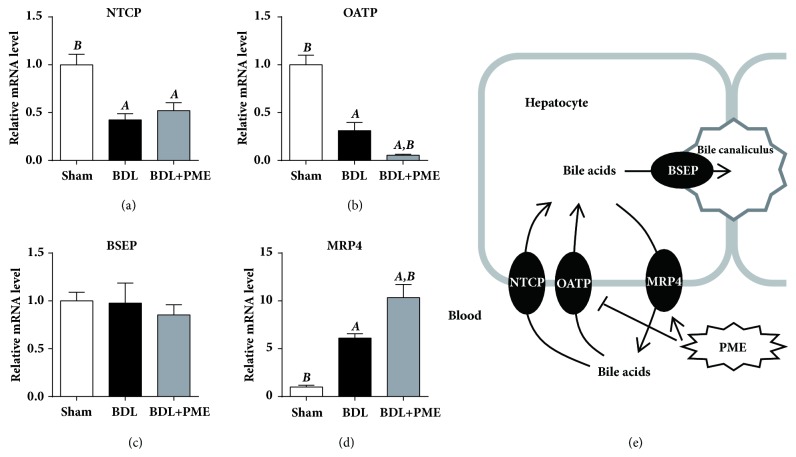
**Effects of PME on hepatic bile transporter mRNA expressions**. Real-time PCR levels of NTCP (a), OATP (b), BSEP (c), and MRP4 (d). mRNA expressions in liver tissues were measured 14 days after sham or BDL. All the levels were equalized to GAPDH and are relative to the level of the sham group. ^A^* p* < 0.05 compared with the sham group; ^B^* p* < 0.05 compared with the BDL group;* n* = 3 in sham group;* n* = 5 each in the BDL and BDL+PME groups.

When RNA from liver samples was analyzed, the expression of hepatic bile acid importing channels, the sodium taurocholate cotransporting polypeptide (NTCP), and organic-anion-transporting polypeptide (OATP) had decreased and that of the bile acid exporting channel and multidrug resistance protein 4 (MRP4) had increased in the BDL group compared with those in the sham-operated group. PME treatment significantly reduced OATP and increased MRP4 expressions. No difference was noted in the expression of the bile salt export pump (BSEP) (another bile acid export channel) among the groups (Figures 4(a)-4(d)). This evidence revealed that the protective effects of PME in cholestatic liver injury might be partially through increasing the export and decreasing the import of bile acid into hepatocytes.

## 4. Discussion

**Figure 5:**
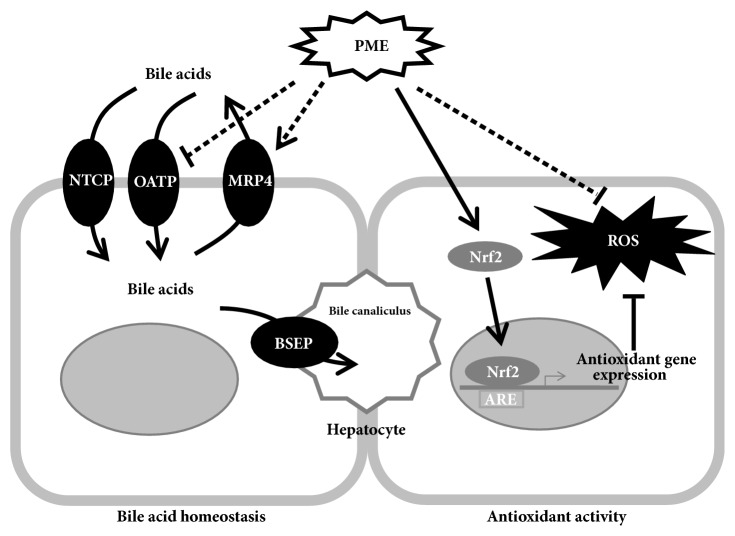
**Proposed hepatoprotective mechanisms of PME**. The current study demonstrated that PME can induce the nuclear translocation of the transcription factor Nrf2, induce ARE-related expression of antioxidant genes, and inhibit ROS production in hepatocyte. In addition, PME also repressed bile acid import and induced basolateral bile acid export under cholestatic liver injury. All these data supported the hepatoprotective activities of PME, including antioxidant activity and bile acid homeostasis.

In this study, our data presented that PME had enhanced hepatoprotective activities in both in vitro and in vivo models. PME induced Nrf2 pathway activation, increased antioxidant-response-element- (ARE-) related gene expressions, and further suppressed H_2_O_2_^−^ and APAP-induced cellular ROS generation in HepG2 cells. PME pretreatment in the APAP-induced mouse liver injury model reduced the severity of liver injury and increased the survival rate. Moreover, PME treatment after BDL also attenuated extrahepatic cholestatic liver injury and may have contributed to reducing hepatic bile acid accumulation. These data support the hepatoprotective activity of PME on redox status and bile acid homeostasis (Figure 5).

Although APAP is a safe drug at a normal therapeutic dose, the possibility of hepatotoxicity still persists. APAP-induced liver injury is one of the best characterized systems of xenobiotic-induced liver injury for evaluating new treatment approaches [14]. After a therapeutic dose, APAP is mainly metabolized and changed into pharmacologically inactive glucuronide and sulfate conjugates, whereas a minor fraction is oxidized into a toxin that binds to the sulfhydryl group of glutathione (GSH) to form an APAP–GSH conjugate that is then excreted in the urine [15]. Excessive N-acetyl-p-benzoquinone imine formation depletes cellular GSH levels, leading to subsequent ROS-mediated lipid peroxidation and liver injury [2, 16]. APAP-mediated liver injury is exacerbated in Nrf2-deficient animals due to reduced expressions of antioxidant genes [17] and the efflux of APAP metabolites [18]. However, liver-specific kelch-like ECH-associated protein 1 (Keap1)-deficient animals were more resistant to APAP-induced liver injury [19]. In addition, constant Nrf2 activation increased the basal hepatic GSH levels and accelerated their recovery after APAP treatment [20]. Recent data also support alleviation of APAP-induced liver injury through activation of the Nrf2 antioxidant pathway [21, 22]. In the current study, PME induced activation of the Nrf2 pathway and decreased ROS production in vitro. In addition, PME also significantly improved the survival of mice with APAP-induced acute liver injury. Pretreatment with PME ameliorated APAP-induced liver injury, as evidenced by reliving increased plasma ALT and AST activities, and by attenuation of hepatic histological changes. Based on these findings, PME showed potential activities against APAP-induced hepatotoxicity by activating Nrf2, upregulating antioxidant defense, and reducing ROS production.

Cholestasis is caused by a reduction in bile flow; it dramatically increases the acid levels of both the liver and serum bile and thereafter may lead to acute liver toxicity, proliferation of bile ducts, and eventual cirrhosis [23]. Because of its regulation of antioxidant and detoxifying abilities, activation of the Nrf2 pathway can be considered a protective mechanism in response to cholestatic liver injury. Changes in bile acid transporters clearly play important roles in controlling the hepatic excretion and subsequent enterohepatic circulation of bile acids, thus influencing the pathophysiology of cholestatic liver injury [24]. Following BDL, although there were no obvious effects on liver injury, Nrf2-deficient animals exhibited increased intrahepatic accumulation of toxic bile acids [25]^.^ Constant activation of Nrf2 by disruption of the Keap1 gene increased MRP efflux transporters, detoxifying enzymes, and antioxidant genes in the liver [26]. A plant-derived triterpenoid, oleanolic acid, also showed the protective effects by ameliorating both lithocholic acid- and BDL-induced cholestatic liver injury through Nrf2-mediated upregulation of MRP efflux transporters [27, 28]. The results of the current study showed that PME can attenuate BDL-induced increases in ALT and AST levels but not total bilirubin or ALP levels. The data implied that the reduction in liver damage due to PME is not mediated by removal of bile duct blockage. From an analysis of mRNA expression, BDL reduces vectorial transporter (NTCP and OATP) expressions and increases the expression of the basolateral exporter, MRP4. PME treatment in BDL animals further reduced OATP and increased MRP4 expressions. These data were further supported by MRP4-deficient mice, which showed an impaired cytoprotective response in obstructive cholestasis [29]. All this evidence suggests that PME treatment induces an adaptive mechanism that protects against cholestatic liver injury by repressing bile acid import and inducing basolateral bile acid export.

Several clinical reports of PM-induced liver injury were noted; however, there is a lack of research fully explaining which ingredient is the most important for liver toxicity, and the underlying mechanisms are unclear. Raw PM exhibited more toxic effects than processed PM did [30]. This raises the possibility that processing alters the properties and functions of remedies; most chemical compositional changes in herbal medicine occur during processing. Ma et al. indicated that the frequency of the cytochrome P450 (CYP) 1A2 1C mutation in Chinese patients with PM-induced acute liver injury was higher than in healthy controls [31]. In addition, the aqueous PM extract induced hepatotoxicity in rats only in combination with the inhibition of CYP1A2 and CYP2E1 activities [32]. This implies that CYP enzymes mediate PM metabolism; however, individuals with these pathogenic single-nucleotide polymorphisms may also have increased susceptibility to drug-induced liver injury, which may be less directly related to the potential toxicity of PM. Xu et al. also reported that THSG exacerbates APAP-induced hepatotoxicity by inducing hepatic expressions of CYP2E1, CYP3A4, and CYP1A2. However, THSG alone showed no hepatotoxicity, but only higher dosages of THSG (>200 mg/kg) enhanced APAP-induced liver injury [33]. Therefore, the extraction methods, genetic susceptibility, and dosage may all be important factors in PM-induced hepatotoxicity.

## 5. Conclusions

Overall, our current study demonstrated that PME exhibited hepatoprotective ability through Nrf2 activation and modulated hepatic bile acid homeostasis in obstructive cholestasis. PME can be used as a potential candidate regimen for managing liver disease.

## Figures and Tables

**Table 1:** Primer sequences.

**Target gene**	Species	**Forward (5**′**-3**′**)**	Reverse (5′-3′)
HO-1	human	ATTCT CTTGG CTGGC TTCCT	CCCCT CTGAA GTTTA GGCCA
NQO1	human	TCCCA GGTTC CAGCA ATTCT	CACTT TGGGA GGCTG AGGTA
GCLc	human	GGAAG TGGAT GTGGA CACCA GA	GCTTG TAGTC AGGAT GGTTT GCG
GAPDH	human	CAGCA AGAGC ACAAG AGGAA G	TGGTA CATGA CAAGG TGCGG
NTCP	mouse	CCTGA TGCCT TTCAC TGGCT TC	GGATG GTAGA ACAGA GTTGG ACG
BSEP	mouse	CCTTG GTAGA GAAGA GGCGA CA	ATGGC TACCC TTTGC TTCTG CC
MRP4	mouse	CACTC AGGAA ACGAA CCTTC TCC	TTGCA CTGCC TGCGT GTTCT CT
OATP1	mouse	GCTGT TCAGT CTTAC GAGTG TGC	CAAGG CATAC TGGAG GCAAG CT
GAPDH	mouse	TCCAC TCACG GCAAA TTCAA C	TCCAC GACAT ACTCA GCACC

## Data Availability

The data used to support the findings of this study are available from the corresponding author upon request.
